# Trends in working conditions and health across three cohorts of older workers in 1993, 2003 and 2013: a cross-sequential study

**DOI:** 10.1186/s12889-019-7736-7

**Published:** 2019-10-26

**Authors:** M. van der Noordt, H. J. Hordijk, W. IJzelenberg, T. G. van Tilburg, S. van der Pas, D. J. H. Deeg

**Affiliations:** 1 Amsterdam UMC, Department of Epidemiology and Biostatistics, Amsterdam Public Health Research Institute, Vrije Universiteit Amsterdam, De Boelelaan 1089 A, 1081 HV Amsterdam, the Netherlands; 2 Department of Health Sciences, Amsterdam Public Health Research Institute, Faculty of Sciences, Vrije Universiteit Amsterdam, Amsterdam, the Netherlands; 3 Department of Sociology, Faculty of Social Sciences, Vrije Universiteit Amsterdam, Amsterdam, the Netherlands; 4 Faculty of Social Work and Applied Psychology, University of Applied Sciences Leiden, Leiden, the Netherlands; 5 Faculty of Governance and Global Affairs, Leiden University, Leiden, the Netherlands

**Keywords:** Working conditions, Physical functioning, Cognitive functioning, Psychological functioning, Cohorts, Older workers

## Abstract

**Background:**

Over the past decades, the number of older workers has increased tremendously. This study examines trends from 1993 to 2013 in physical, cognitive and psychological functioning among three successive cohorts of Dutch older workers. The contribution of the changes in physical and psychosocial work demands and psychosocial work resources to change in functioning is examined. Insight in health of the older working population, and in potential explanatory variables, is relevant in order to reach sustainable employability.

**Methods:**

Data from three cohorts (observations in 1993, 2003 and 2013) of the Longitudinal Aging Study Amsterdam (LASA) were used. Individuals aged 55–65 with a paid job were included (*N* = 1307). Physical functioning was measured using the Timed Chair Stand Test, cognitive functioning by a Coding Task and psychological functioning by the positive affect scale from the CES-D. Working conditions were deduced from a general population job exposure matrix. Linear and logistic regression analyses were performed.

**Results:**

From 1993 to 2013, time needed to perform the Timed Chair Stand Test increased with 1.3 s (95%CI = 0.89–1.71), to a mean of 11.5 s. Coding Task scores increased with 1.7 points (95%CI = 0.81–2.59), to a mean of 31 points. The proportion of workers with low positive affect increased non-significantly from 15 to 20% (*p* = 0.088). Only the improvement in cognitive functioning was associated with the change in working conditions. The observed decrease of physically demanding jobs and increase of jobs with higher psychosocial resources explained 8% of the improvement.

**Conclusions:**

Changes in working conditions may not contribute to improved physical and psychological functioning, but do contribute to improved cognitive functioning to some extent. Further adjustment of physical work demands and psychosocial work resources may help to reach sustainable employability of older workers.

## Background

Over the last decades, there have been demographic and work environment changes that may have influenced health of the older working population in Western countries. First, as a result of the ageing population and consequential need of prolonging working lives, employment rates of older people have increased. Second, due to technological developments and a transition towards a more service-based society, less jobs are physically demanding and more jobs require cognitive and communicative abilities [1–4]. Generally, working conditions impact physical, cognitive and psychological health, and this impact might be greater for older workers as they need more time to recover from physically or psychosocially demanding tasks than younger workers [5–8]. Insight in the health trends of older workers is relevant in order to reach sustainable employability of older workers [9]. We study the change in physical, cognitive and psychological functioning of older workers aged 55 to 65 over twenty years and whether this change is related to change in working conditions.

To assess working conditions, we investigate three types of working conditions that fit into the job demands-resources model: physical demands, psychosocial demands and psychosocial resources [10]. Physical demands are for example the necessity to use force or to work in uncomfortable positions [11, 12]. Psychosocial demands are for example high time pressure or high cognitive demands [11–13]. Psychosocial resources are for example job autonomy (i.e. the ability to make decisions regarding work), which help the worker in reducing work demands, achieving work goals and personal development [12, 14].

Evidence shows that these working conditions are associated with a variety of health domains such as physical, cognitive and psychological functioning. High physical demands, such as heavy lifting, impair physical functioning [6]. High psychosocial demands, such as long working hours, work overload and pressure, negatively affect psychological functioning [8, 15, 16]. In contrast, psychosocial demands are associated with better cognitive function in middle and later age [7]. High psychosocial resources, such as job autonomy, counterbalance the effects of physical and psychosocial demands on physical, psychological and cognitive functioning, and are associated with improvement in cognitive and psychological functioning [7, 8, 10, 15].

In many European countries, working conditions have changed over the past 20 years. Physical demands have decreased [2, 3], while psychosocial demands, e.g. work intensity and cognitive demands, have increased [2–4, 17] and psychosocial resources, e.g. autonomy at work, have also increased [17].

Previous studies examining health trends show that physical and psychological functioning deteriorated and cognitive functioning improved across successive cohorts of older adults over the past 20 years [18–23]. Explanations for these changes are increased prevalence of specific diseases, such as arthritis and diabetes mellitus, and obesity [20, 21] and increased educational level [18], respectively. The contribution of changes in working conditions to health trends across cohorts of older adults has not been studied yet.

This study examines trends in physical, cognitive and psychological functioning across three successive cohorts of Dutch workers aged 55 to 65 in 1993, 2003 and 2013. The contribution of the changes in physical and psychosocial demands and psychosocial resources to change in functioning is examined. First, we expect a deterioration of physical functioning, which is counterbalanced by the change in working conditions (Hypothesis 1a & 1b). Second, we expect an improvement in cognitive functioning, which is partly attributed to the change in working conditions (Hypothesis 2a & 2b). Third, we expect a deterioration of psychological functioning, which is not affected by the change in working conditions because of the counterbalancing effects of increased psychosocial demands on the one hand, and increased psychosocial resources on the other hand (Hypothesis 3a & 3b).

## Methods

### Study design and sample

This study has a cross-sequential design. It uses data from the Longitudinal Aging Study Amsterdam (LASA), an ongoing study of physical, emotional, cognitive and social functioning of older adults age 55 and above in the Netherlands [24, 25]. Data from the three cohorts, collected in 1993, 2003 and 2013, were used. Individuals aged 55 to 65 years with paid work ≥1 h per week were included. The cut-off point of 1 h ensures that all types of jobs, including part-time, temporary or seasonal, are taken into account because the corresponding working conditions of all these types of jobs may have influenced health [26]. This sample of workers consisted of 1307 respondents (cohort 1, 1993: *n* = 274; cohort 2, 2003: *n* = 416; cohort 3, 2013: *n* = 617). In addition, a same aged sample of non-working individuals was used for a comparison of the health trends (*n* = 672; *n* = 577; *n* = 374, respectively).

### Outcome variables

#### Physical functioning

Physical functioning was measured by the Timed Chair Stand Test, involving standing up without the use of arms five times as quickly as possible. Time in seconds was recorded and used as a continuous variable. Participants who were not able to perform the test (*n* = 16), who performed the test incorrectly (*n* = 18), who performed the test in > 25 s (*n* = 3) and who had not performed the test for unknown reasons (*n* = 22), were excluded. Physical performance tests are good predictors of morbidity, hospitalisation and death [27]. The Timed Chair Stand Test has been shown to be a valid and reliable measure for functional mobility in a sample of older females [28].

#### Cognitive functioning

Cognitive functioning was measured by an adjusted version of the Alphabet Coding Task-15. The Coding Task involves a form given to the participant, showing two rows of characters. Each character in the upper row belongs to a character in the bottom row. The participant is asked to make as many combinations as possible. This is done in three cycles of 1 min. The mean score of the three cycles was calculated and used as a continuous variable [29]. Participants who had not participated in the medical interview (*n* = 135) or had not executed the Coding Task for other reasons (*n* = 5) were excluded. The Coding Task is believed to reflect various processes such as attention processes, memory function, and perceptual organisation and speed, but its validity has not been assessed [30].

#### Psychological functioning

Psychological functioning was measured by positive affect, a subscale of the Center for Epidemiologic Studies Depression Scale (CES-D) [31]. Positive affect was chosen, because we expected that more variation would be found when using it instead of the full CES-D [32]. Cronbach’s alpha of positive affect (items 4, 8, 12 and 16) in our sample was 0.71. Items have four response categories, ranging from (0) ‘rarely to never’ to (3) ‘mostly or always’. Items scores were summed. The variable was dichotomised because of a right-skewed distribution of the continuous variable, with the cut-off set at the first quartile to identify workers with low positive affect. Twenty-three respondents were excluded because they had not (fully) responded to the CES-D for unknown reasons.

### Main determinants

#### Working conditions

Working conditions of the current job were deduced from the general population job exposure matrix (GPJEM) for 55 to 65 year olds [12]. The GPJEM indicates levels of exposure probability of physical and psychosocial demands and psychosocial resources, based on job category. Physical demands involve the necessity to use force during work, working in uncomfortable positions, and performing repetitive movements. Psychosocial demands involve time pressure, task requirements, and cognitive demands. Psychosocial resources involve job autonomy and variation in activities at work. Working conditions scores range from low to high exposure probability (physical demands: 0–4; psychosocial demands: 0–6; psychosocial resources: 0–4) [12].

### Covariates

Covariates used in this study were chosen based on three criteria, of which at least two should be met. First, the covariate is expected to have changed in the (working) population over the past two decades. Second, the covariate is expected to be associated with working conditions. Third, the covariate is expected to be associated with one of the health outcomes studied. The following covariates were therefore included: sex, age, education level, partner status, chronic diseases, alcohol use, smoking, BMI, physical activity, working hours and mastery. Sex and date of birth were obtained from municipal registries. The highest educational level completed categorised into low, intermediate and high, according to the International Standard Classification of Education [33]. Partner status was measured by the question: “Are you currently living with someone, whom you consider to be your partner?”. Self-report of chronic diseases was categorised into having 0, 1 or >  1 chronic diseases. Respondents were asked for the presence of a chronic diseases in one question with the following answering options: 1) chronic non-specific lung disease 2) cardiac disease, 3) peripheral arterial disease, 4) diabetes mellitus, 5) cerebrovascular accident or stroke, 6) osteoarthritis, 7) rheumatoid arthritis, 8) cancer and 9) other. The self-reports on chronic diseases are fairly accurate when compared to general practitioner information [34]. Self-rated health was measured by a single self-report question: “How is your health in general?”, with five response categories, dichotomised into good versus less than good health [35]. Alcohol use was based on the Garretsen index [36], which categorises alcohol use into light, moderate, and excessive. Smoking was categorised into never smoked, smoked ≥15 years ago, smoked < 15 years ago, and currently smoking [37]. Body Mass Index (BMI) was calculated from measured height and weight and was divided in the categories normal weight (BMI <  25), overweight (25 ≤ BMI < 30) and obese (BMI ≥ 30) [37]. Regarding physical activity, the number of minutes per day were calculated based on the frequency and duration of all types of physical activity performed in the previous 2 weeks, i.e. walking outdoors, biking, gardening, light household activities, heavy household activities and two of their most frequently performed sports activities [38]. The number of working hours per week was used as a continuous variable. Working hours above 80 were adjusted to 80 h. Mastery, i.e. the extent to which a person perceives himself or herself to be in control of events and ongoing situations, was used as a continuous variable measured by the Pearlin Mastery scale [39].

### Data analyses

#### Multiple imputation

Of the respondents 14% had at least one missing value on the working conditions or covariates. Multiple imputation was performed to make inferences based on all variables described above (except for self-rated health). A pooled sample of twenty imputations was used.

#### Health trends

**Fig. 1:**
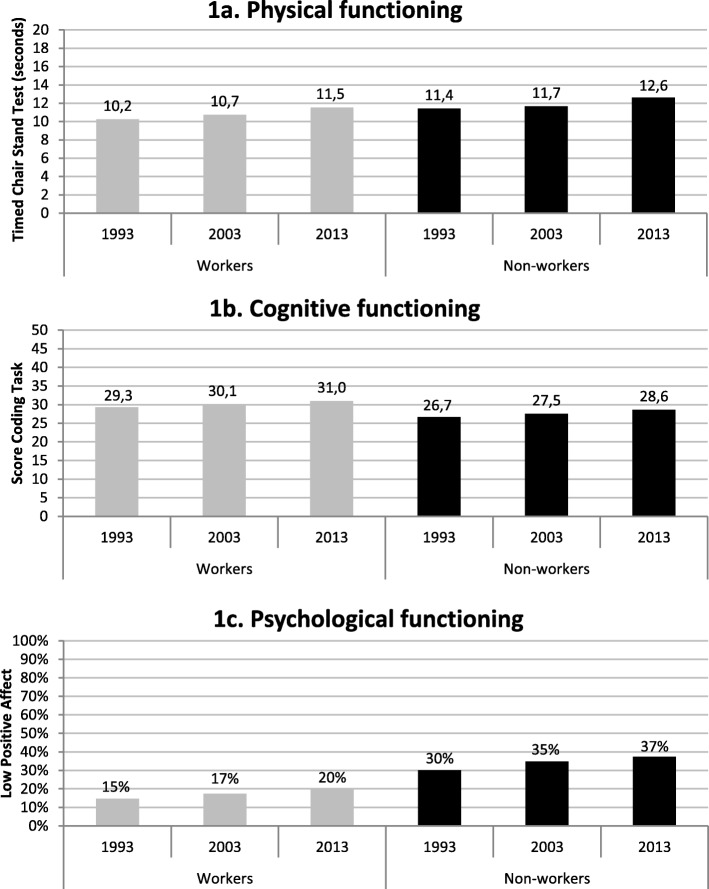
Trends in health among successive cohorts of workers and non-workers, adjusted for sex and age. Note: **a** Workers *n* = 1248; Non-workers *n* = 1474. **b** Workers *n* = 1167; Non-workers *n* = 1443. **c** Workers *n* = 1284; Non-workers *n* = 1565

For each health outcome trends were analysed using linear regression analyses adjusted for sex and age (in quartiles). First, the association between cohort number and the health outcomes were analysed for workers and non-workers separately, and means and proportions are presented in Fig. 1a-c. Second, these trends in health functioning were analysed for the total sample of workers and non-workers, with an interaction term between cohort number and work status to examine whether the trends differed between workers and non-workers. Third, differences in health functioning between workers and non-workers in each cohort separately were assessed.

#### The contribution of working conditions

The contribution of working conditions to the health trends were analysed using linear and logistic regression (Table 2). Multiple models were assessed for each health outcome. In Model 1, the relationship between cohort number and health outcomes was examined adjusted for sex and age. In Model 2, relevant covariates were included. In Model 3 (a/b/c), working conditions were added separately. In Model 4, working conditions were added together. Tolerance appeared < 0.50 for psychosocial demands and resources, which correlated highest with physical demands. New variables were constructed for psychosocial demands and resources, in which the residuals of physical demands were removed, and were used in Model 4. The effect sizes of dummy variables for cohort 2003 and 2013 in Model 2 were compared to Model 1, and in Model 3 and 4 to Model 2, to examine the contribution of the trend in covariates and working conditions, respectively, on the trend in health functioning. If one of the effect sizes of dummy variables cohort 2003 or 2013 increased or decreased more than 10%, we considered it as relevant suppressor(s) or explanatory variable(s). All analyses were performed in SPSS 22. The assumptions of regression analyses were checked and proved to be met.

### Sensitivity analysis

We compared self-rated health of respondents with and without missing values on the health outcomes, using chi-square analyses. If the excluded respondents were in poorer self-rated health, we examined whether the proportion of excluded respondents differed between the cohorts. Self-rated health was chosen because it has few missing values (*n* = 1) and it has been shown to be associated with physical, cognitive and psychological functioning [35, 40].

## Results

### Study characteristics

In Table 1, basic characteristics of Dutch workers aged 55 to 65 are shown within each cohort. Over the successive cohorts, the proportion of workers increased from 29 to 62%. The proportion of women increased among the workers (from 36 to 46%). Mean age decreased slightly at first, and increased subsequently, indicating that in 2003 in particular the proportion of 55–60-year-olds has increased, and in 2013 the proportion of 60–65-year-olds. Mean exposure to physical demands decreased, and exposure to psychosocial demands and psychosocial resources increased. This is a result of a change from sectors representing manual labour to sectors representing office jobs.

**Table 1:** Descriptive statistics of successive cohorts of older workers, *N* = 1307

		Cohort 1: 1992/1993 (*N* = 274)	Cohort 2: 2002/2003 (*N* = 416)	Cohort 3: 2012/2013 (*N* = 617)	*P*-value^a^
% of total cohort		29%	42%	62%	< 0.001
Basic demographics					
Sex	Males	174 (64%)	253 (61%)	336 (54%)	0.019
	Females	100 (36%)	163 (39%)	281 (46%)	
Age	Mean	58.9 (2.7)	58.6 (2.6)	59.4 (2.6)	< 0.001
Working conditions					
Physical demands	Mean score (0–4)	2.1 (1.6)	1.7 (1.6)	1.4 (1.6)	< 0.001
Psychosocial demands	Mean score (0–6)	1.1 (1.9)	1.5 (2.0)	2.0 (2.1)	< 0.001
Psychosocial resources	Mean score (0–4)	1.1 (1.3)	1.5 (1.4)	1.8 (1.5)	< 0.001
Occupational sector	Administrative/commercial	74 (27%)	110 (27%)	142 (23%)	< 0.001
	General	20 (7%)	26 (6%)	43 (7%)	
	Pedagogical	19 (7%)	34 (8%)	68 (11%)	
	Agricultural	27 (10%)	20 (5%)	18 (3%)	
	Natural science	0 (0%)	1 (0%)	23 (4%)	
	Technical	53 (19%)	91 (22%)	81 (13%)	
	Transport	11 (4%)	19 (5%)	40 (6%)	
	(Para)medical	11 (4%)	19 (5%)	46 (7%)	
	Juridical/security	6 (2%)	15 (4%)	26 (4%)	
	Cultural/linguistic	4 (2%)	12 (3%)	28 (5%)	
	Social science	9 (3%)	18 (4%)	20 (3%)	
	Care services	34 (12%)	44 (11%)	58 (9%)	
	Management	5 (2%)	6 (1%)	23 (4%)	
Covariates					
Education	Low	153 (56%)	204 (49%)	225 (36%)	
	Intermediate	65 (24%)	81 (19%)	164 (27%)	< 0.001
	High	56 (20%)	131 (31%)	228 (37%)	
Partner status	No partner	47 (17%)	70 (17%)	102 (17%)	
	Partner	227 (83%)	346 (83%)	515 (83%)	0.973
Working hours per week	Mean (range 1–80)	32.7 (18)	30.3 (16)	31.1 (14)	0.094
Self-reported chronic diseases	0	125 (46%)	144 (35%)	169 (27%)	
	1	92 (34%)	150 (36%)	203 (33%)	< 0.001
	> 1	57 (21%)	122 (29%)	245 (40%)	
Mastery	Mean	18.7 (3.0)	18.9 (3.0)	19.1 (3.0)	0.141
Alcohol use	Does not drink	23 (8%)	22 (5%)	60 (10%)	
	Non-excessive	224 (82%)	335 (81%)	503 (82%)	0.009
	(Very) excessive	27 (10%)	59 (14%)	54 (9%)	
Smoking	Never smoked	58 (21%)	86 (21%)	163 (26%)	
	Smoked ≥15 years ago	61 (22%)	130 (31%)	248 (40%)	< 0.001
	Smoked < 15 years ago	61 (22%)	68 (16%)	101 (24%)	
	Currently smoking	94 (34%)	132 (32%)	105 (17%)	
BMI	Normal (< 25)	98 (36%)	124 (30%)	217 (35%)	
	Overweight (25–30)	144 (53%)	203 (49%)	265 (43%)	0.003
	Obesity (≥ 30)	32 (12%)	89 (21%)	135 (22%)	
Physical activity	Mean (min/day)	147 (120)	133 (106)	132 (104)	0.138^b^

^a^On Pearson chi-square test or oneway ANOVA

^b^*P*-value of log-transformed means = 0.802

### Physical functioning

Figure 1a shows that physical functioning of successive cohorts of workers deteriorated over time, supporting Hypothesis 1a. In 1993, workers performed the Timed Chair Stand Test on average in 10.2 s and in 2013 in 11.5 s (*p* < 0.001). Among non-workers, this is 11.4 and 12.6 s, respectively (*p* < 0.001). The interaction term between cohort number and work status is insignificant, implying that there is no difference in the trends between workers and non-workers (*p* = 0.411). In each cohort, physical functioning is better among workers compared to non-workers (*p* < 0.05).

Among workers, the deterioration in physical health of the cohorts is also shown in the first column of Table 2 (Model 1). The effect of cohort number on physical health remains significant after adjustment for covariates (Model 2). Working conditions do not affect the deterioration in physical functioning, in contrast to Hypothesis 1b (see Model 3a/b/c for the contribution of the separate types of working conditions and Model 4 for the joint model).

**Table 2:** Trends in health among successive cohorts of older workers

		Physical functioning *N* = 1248	Cognitive functioning *N* = 1167	Psychological functioning *N* = 1284
		B (95% C.I.)	B (95% C.I.)	OR (95% C.I.)
Model 1	Cohort (ref. 1993)			
	2003	0.51 (0.07–0.94)*	0.79 (−0.16–1.73)	1.21 (0.80–1.82)
	2013	1.30 (0.89–1.71)***	1.70 (0.81–2.59)***	1.39 (0.95–2.04)
Model 2	Cohort (ref. 1993)			
	2003	0.50 (0.06–0.94)*	0.36 (− 0.53–1.26)	1.17 (0.74–1.85)
	2013	1.25 (0.82–1.67)***	0.66 (− 0.21–1.54)	1.39 (0.90–2.14)
Model 3a	Cohort (ref. 1993)			
	2003	0.52 (0.08–0.96)*	0.28 (−0.60–1.17)	1.18 (0.75–1.87)
	2013	1.28 (0.85–1.71)***	0.49 (−0.37–1.36)	1.41 (0.91–2.18)
	Working conditions			
	Physical demands (0–4)	0.09 (−0.02–0.21)	− 0.66 (− 0.89--0.43)***	1.04 (0.93–1.17)
Model 3b	Cohort (ref. 1993)			
	2003	0.50 (0.07–0.94)*	0.36 (−0.54–1.25)	1.17 (0.74–1.85)
	2013	1.26 (0.83–1.69)***	0.65 (−0.23–1.52)	1.39 (0.90–2.14)
	Working conditions			
	Psychosocial demands (0–6)	−0.04 (− 0.13–0.06)	0.07 (− 0.13–0.26)	1.02 (0.93–1.12)
Model 3c	Cohort (ref. 1993)			
	2003	0.50 (0.06–0.94)*	0.29 (−0.61–1.18)	1.19 (0.75–1.88)
	2013	1.24 (0.82–1.67)***	0.54 (−0.34–1.42)	1.42 (0.92–2.19)
	Working conditions			
	Psychosocial resources (0–4)	0.02 (−0.11–0.15)	0.35 (0.08–0.63)*	0.93 (0.82–1.06)
Model 4	Cohort (ref. 1993)			
	2003	0.50 (0.06–0.94)*	0.29 (−0.60–1.17)	1.20 (0.76–1.90)
	2013	1.26 (0.83–1.68)***	0.52 (−0.35–1.39)	1.43 (0.92–2.21)
	Working conditions			
	Physical demands (0–4)	0.09 (−0.03–0.21)	− 0.62 (− 0.86--0.38)***	1.04 (0.92–1.17)
	Psychosocial demands (0–6)	−0.05 (− 0.16–0.06)	−0.10 (− 0.33–0.12)	1.06 (0.95–1.18)
	Psychosocial resources (0–4)	0.14 (− 0.03–0.31)	0.10 (− 0.34–0.36)	0.91 (0.76–1.08)

Model 1. Model adjusted for sex and age

Model 2. Model 1 + educational level, partner status, chronic diseases, alcohol use, smoking, BMI, physical activity, working hours, mastery

Model 3. a. Model 2 + physical demands; b. Model 2 + psychosocial demands; c. Model 2 + psychosocial resources

Model 4. Model 2 + physical demands, psychosocial demands and psychosocial resources

*: *p* < 0.05; ***: *p* < 0.001

### Cognitive functioning

The cognitive functioning of successive cohorts of workers improved over time, supporting Hypothesis 2a (Fig. 1b). Among workers, the mean coding task score increases from 29.3 in 1993 to 31.0 in 2013 (*p* < 0.001). Among non-workers, it increases from 26.7 to 28.6 (*p* < 0.001) over the same period. These trends do not differ significantly (interaction term: *p* = 0.558). In each cohort, cognitive functioning is better among workers compared to non-workers (*p* < 0.001).

The improvement in cognitive functioning among successive cohorts of workers can for 61% be explained by the covariates added in Model 2 (Table 2). In particular the rise in educational level explains this improvement (result not shown in Table). The change in working conditions explains it also partly, given the decrease of > 10% in B for cohort 2013, from 0.66 in Model 2 to 0.52 in Model 4, supporting Hypothesis 2b. After inclusion of the other covariates, change in working conditions explained another 8% of the change in health from cohort 1 to cohort 3. In particular, the decrease of physically demanding jobs (Model 3a) and increase of jobs with high psychological resources (Model 3c) contributed.

### Psychological functioning

The proportion of workers with low positive affect rises non-significantly (15% in 1993 and 20% in 2013; *p* = 0.088), providing no sufficient support to Hypothesis 3a (Fig. 1c). The proportion of non-workers with low positive affect increases (30% in 1993 to 37% in 2013; *p* < 0.001). These trends do not differ significantly (interaction term: *p* = 0.631). In each cohort, psychological functioning is better among workers compared to non-workers (*p* < 0.001).

The non-significant increase of the proportion of workers with low positive affect is not related to change in covariates (Model 2, Table 2), and neither to the change in working conditions (Model 3a/b/c and Model 4), in contrast to Hypothesis 3b.

### Sensitivity analysis

Workers with missing values on physical and cognitive functioning were in poorer self-rated health compared to workers with no missing values (*p* > 0.001 and *p* > 0.01, respectively). There was no difference in self-rated health between workers with and without data on psychological functioning (*p* = 0.668). Missing values regarding physical functioning were relatively evenly distributed over the three cohorts. Missing values regarding cognitive functioning were most prevalent in cohort 2003.

## Discussion

This study assesses trends in physical, cognitive and psychological functioning among Dutch workers aged 55 to 65 from 1993 to 2013. In particular, the contribution of the decrease in physical work demands and increase of psychosocial work demands and resources to these trends are examined.

The findings show that physical functioning deteriorated across three successive cohorts of older workers, which supports Hypothesis 1a. Although there are no validated cut-off points for the Timed Chair Stand Test to determine the severity of functional mobility, we consider the increase of 1.3 s to a mean of 11.5 s for workers to perform the Timed Chair Stand Test as substantial. In comparison, another study shows that the mean score for healthy subjects was 12.5 s and for subjects with high risk of fallings, 14.8 s (age category 74–98) [41].

Hypothesis 1b, stating that the decline in physical functioning is counterbalanced by the change in working conditions, is not supported. There is no association between working conditions and physical functioning at all. Potentially, the beneficial effects of the decrease in physical demanding jobs were cancelled out by the negative effects of the increase of non-physical demanding jobs, in which sedentary behaviour is common and also negatively affects physical functioning [42]. Another explanation is that the Timed Chair Stand Test does not cover all aspects of physical functioning, as especially the lower limbs are tested [43]. A systematic review showed that there is reasonable evidence for an association between physically demanding work and work-related musculoskeletal disorders. However, this applies only to neck, low back, upper limb and hip disorders. There was no or insufficient evidence for non-specific lower limb and knee disorders [6].

Hypothesis 2a and 2b, stating that cognitive functioning will improve and that this change can partly be attributed to the change in working conditions, are supported. Remarkably, the decrease of physically demanding jobs and increase of jobs with psychosocial resources contributed particularly, while previous research shows that in particular psychosocial demands are associated with cognitive functioning [7]. Naturally, the decrease of physically demanding jobs and increase of jobs with high psychosocial resources is also accompanied by an increase of jobs with cognitive demands. The improvement in cognitive functioning is also explained by the rise in educational level, in accordance with results from previous research [18].

Hypothesis 3a, stating that psychological functioning will deteriorate over the successive cohorts, is not supported by our study. Although the increased prevalence of low positive affect from 15 to 20% is not significant in our sample of workers, an increase of five percentage points is considered relevant given that workers with psychological health problems have lower workability and productivity [44].

Hypothesis 3b, stating that the deterioration in psychological functioning was not affected by the change in working conditions, was supported. This was hypothesized because the negative effect of increased psychosocial work demands was expected to be counterbalanced by the positive effect of increased psychosocial work resources. However, in contrast to previous studies [8, 15, 16], these two types of working conditions separately did not affect psychological functioning. Potentially, the expected effect of the change in working conditions was overestimated. One of the systematic reviews that found ‘moderate’ evidence for an association between psychosocial work demands and psychological functioning stressed that there was an indication of publication bias resulting in an overestimated association [16].

### Methodological considerations

The use of LASA data provides the unique possibility to compare three large cohorts over 20 years. In addition, LASA contains a sample of the Dutch older population, which may decrease the risk of selection bias. Data on the same tests and questionnaires were available for each of the cohorts. This made it possible to study trends and the contribution of covariates to these trends.

A second strength of this study is the choice for objective health measures from the LASA data [24, 25]. This decreases the chance of information bias and such measures can be sensitive to changes the respondent does not perceive yet [27].

There are also a number of limitations. A first limitation is that the GPJEM has only partly been validated. Rijs et al. examined the association between the GPJEM and health, but research on the association between the GPJEM and self-reported working conditions should still be performed [12]. In addition, the GPJEM does not take heterogeneity within job categories into account, because information is aggregated [12]. This limitation might have biased our findings toward the null.

Second, we used data on the current job. Workers with reduced functioning may have already switched their job because they were no longer able to perform high physical or psychosocial demanding tasks, and other, better fitting jobs were available. Data on the longest job was only available in cohort 1. It showed that only a small minority of the respondents reported a different longest job compared to the current job, and the associated working conditions remained on average the same. Therefore, we do not expect bias because of this.

Third, reversed causality may have played a role. Our findings indicate that the change in working conditions contributed to the improvement in cognitive functioning. However, such improvement may cause an increased interest from workers to perform jobs with high psychosocial demands and resources. In favour of the first, a systematic review based on longitudinal studies showed that high psychosocial demands and resources at one point in time were prospectively associated with higher levels of cognitive function in midlife and late life [7].

Fourth, workers in poor self-rated health had more often missing data regarding cognitive functioning, and were therefore excluded from the analyses. They were also more prevalent in cohort 2003, than in cohort 1993 and 2013. Still, we do not expect bias from this because the proportion of workers with missing data regarding cognitive functioning was low (< 2%).

### Implications for practice and further research

This study shows a significant deteriorating trend in physical functioning and non-significant deteriorating trend in psychological functioning among workers from 1993 to 2013. The change in working conditions hardly contributed to these observed trends. Taking into account that only since 2012 the statutory retirement age has increased [45], this suggests that without any interventions, future generations of older workers will be less healthy in physical and psychological sense. This may be caused by further deterioration of physical and psychological public health and further increase of the proportion of older adults participating in the workforce. These developments in health may hamper sustainable employability, which is a shared responsibility of workers, employers and policy makers. One aspect is that employers must ensure that the job requirements meet the work capabilities of older workers and support older workers with health issues, to prevent early work exit [46, 47]. More research is needed to examine the optimal work context [9].

This study also shows an improving trend in cognitive functioning among workers from 1993 to 2013, and the change in working conditions contributed to some extent to this improvement. According to a study on labour market forecasting, the number of physically demanding jobs are expected to further decrease, while the proportion of higher education jobs will further increase [48]. Moreover, the educational level of future cohorts of older adults continues to increase as well [49]. Therefore, the job requirements of future jobs are increasingly likely to meet the work capabilities of future older workers. Further adjustment of physical and psychosocial working conditions may help to reach sustainable employability of older workers [15]. However, the proportion of elementary and lower-education jobs is expected to remain stable, with 30% of all jobs [48]. In future research should be searched to effective ways to support these workers in order to reach the retirement age while working in good wellbeing, work ability and productivity [50].

## Conclusions

This study shows that physical and psychological functioning of three successive cohorts of older workers aged 55 to 65 deteriorated and cognitive functioning improved in the period from 1993 to 2013. This change in functioning is largely a reflection of the change in functioning of the total population of 55–65 years-olds. The decrease in physically demanding jobs and increase in jobs with high psychosocial demands and resources hardly contributed to understanding of the observed trends in physical and psychological functioning. Only cognitive functioning benefited to some extent from these changes in working conditions.

## Data Availability

Data from the Longitudinal Aging Study Amsterdam are not publicly available due to grounds of confidentiality and anonymity. Part of the data can be made available upon request, provided that an agreement is made up. Research proposals should be submitted to the research director, prof. dr. M. Huisman (lasa@amsterdamumc.nl); For more information see: www.lasa-vu.nl.

## Abbreviations

BMI: Body Mass Index
CES-D: Center for Epidemiologic Studies Depression Scale
GPJEM: General Population Job Exposure Matrix
LASA: Longitudinal Aging Study Amsterdam
